# Implantation of three transcatheter aortic valves for embolization of two valves caused by under-expansion: a case report

**DOI:** 10.1093/ehjcr/ytaa497

**Published:** 2020-12-15

**Authors:** Masaki Tsuda, Ryu Shutta, Masami Nishino, Jun Tanouchi

**Affiliations:** Division of Cardiology, Osaka Rosai Hospital, 1179-3 Nagasone-cho, Sakai, Osaka, Japan

**Keywords:** Case report, Aortic stenosis, Complication, Valve embolization, Transcatheter aortic valve implantation

## Abstract

**Background:**

Transcatheter aortic valve embolization is one of the serious complications of transcatheter aortic valve implantation (TAVI). We present a case of TAVI that needed implantation of three transcatheter aortic valves owing to the embolization of two self-expandable valves (SEVs).

**Case summary:**

An 88-year-old woman underwent TAVI using a 26-mm SEV. After valve deployment, the SEV embolized to the ascending aorta during the removal of the delivery system (DS) of the SEV (DS-SEV) from the SEV. An additional SEV was implanted, which also embolized upwards. Multi-directional fluoroscopy revealed extreme under-expansion of the second SEV, which caused valve embolization due to catching of the DS-SEVs in the SEVs. Finally, a 23-mm balloon-expandable valve was successfully implanted, which was also under expanded on fluoroscopic assessment. The patient was stable without sequelae at the 1-month follow-up.

**Discussion:**

Pre-procedurally predicting SEV under-expansions was difficult because pre-procedural computed tomography revealed no massive calcification on the aortic valve, and fluoroscopy indicated adequate expansion of the SEVs at the angle where the valves were deployed. We verified the possibility of catching of a DS-SEV in an under-expanded SEV in an *in vitro* test, which showed that the DS-SEV was caught in the extremely under-expanded SEV. Furthermore, balloon dilation might release the catch of the DS-SEV by changing the DS-SEV position. Therefore, we recommend performing multi-directional fluoroscopy to evaluate SEV expansion before DS-SEV removal from an SEV. Furthermore, if catching of a DS-SEV occurs, balloon dilation might be useful for releasing the catch and safely removing the DS-SEV.

**Learning points**

- Under-expansion of a transcatheter aortic valve (TAV) can cause valve embolization due to catching of the delivery system (DS) of the TAV (DS-TAV) in the TAV stent during DS-TAV removal in TAV implantation.
- Confirming the expansion of a TAV using multi-directional fluoroscopy before removing the DS-TAV is important to avoid TAV embolization caused by catching of the DS-TAV in the TAV stent.
- Large-sized balloon dilation at the site where a DS-TAV is caught in the TAV stent might be one of the solutions for preventing TAV embolization due to catching of the DS-TAV in the TAV stent.

## Introduction

Transcatheter aortic valve embolization (TAVE) is a rare but serious complication of transcatheter aortic valve implantation (TAVI) that is associated with high mortality rates.^1^ The common causes of TAVE are sizing errors, post-dilation, and fast-rate pacing failure. However, identifying the cause of TAVE is occasionally difficult.^2^^,^^3^ This report describes a case of TAVI that needed three transcatheter aortic valves [TAV; two self-expandable valves (SEVs) and one balloon-expandable valve (BEV)] because of embolization of two SEVs. The valve embolizations occurred due to catching of the delivery system (DS) of the SEV (DS-SEV) in the SEVs that were extremely under-expanded.

## Timeline

**Table:** 

Time	Events
March 2020	The patient had shortness of breath, classified as New York Heart Association (NYHA) class III.
April 2020	The patient had a possible aortic stenosis due to systolic murmur and was referred to our hospital. Transthoracic echocardiography (TTE) showed left ventricular ejection fraction (LVEF) of 54%, and severe aortic stenosis. The patient’s EuroSCORE II was 5.03%, and our Heart Team considered transcatheter aortic valve implantation (TAVI) to be appropriate because of the patient’s advanced age.
12 May 2020	The patient underwent transfemoral TAVI with a self-expandable valve (SEV). The SEV embolized to the ascending aorta during the removal of the delivery system (DS) of SEV (DS-SEV). The haemodynamics collapsed immediately after the valve embolization, and an emergency veno-arterial extracorporeal membrane oxygenation (V-A ECMO) was introduced. An additional SEV was implanted. However, the SEV also embolized to the ascending aorta because of a catch of the DS-SEV in the SEV. Finally, a balloon-expandable valve was successfully implanted in the native aortic valve. The patient was transferred to the intensive care unit under intubation and the VA-ECMO support.
13 May 2020	The V-A ECMO was removed and extubation was performed because of the patient recovery. Single antiplatelet therapy using clopidogrel (75 mg/day) was administered.
14 May 2020	The patient was moved out of the intensive care unit.
21 May 2020	The patient was discharged to home by herself without further complications.
June 2020	The patient was alive without any sequelae with slight shortness of breath, classified as NYHA class II. TTE showed LVEF of 61% with a mean pressure gradient of 10 mmHg, and trivial aortic regurgitation (paravalvular leakage).

## Case presentation

An 88-year-old woman with a history of hypertension and dyslipidaemia (on telmisartan 20 mg/day and atorvastatin 5 mg/day) was diagnosed with severe aortic stenosis. The patient’s blood pressure, heart rate, and oxygen saturation (room air) were 114/63 mmHg, 82/min, and 94%, respectively. The cardiac symptom of the patient was shortness of breath, classified as New York Heart Association (NYHA) class III. Physical examination revealed normal respiratory sounds, a systolic ejection murmur, and leg oedema. The patient was classified as having class 4 clinical frailty. Electrocardiography revealed normal sinus rhythm and normal atrial-ventricular conduction. Chest radiography showed cardiomegaly and pulmonary congestion with right pleural effusion. Transthoracic echocardiography showed a normal left ventricular ejection fraction (LVEF; 54%) with severe aortic stenosis, a mean pressure gradient of 63 mmHg. The patient had mild aortic and mitral regurgitation. All laboratory values were normal, except for N-terminal pro-brain natriuretic peptide level (4687 pg/mL, normal range <125 pg/mL). The patient’s EuroSCORE II was 5.03%; our Heart Team selected TAVI because of the patient’s advanced age and frailty.

**Figure 1:**
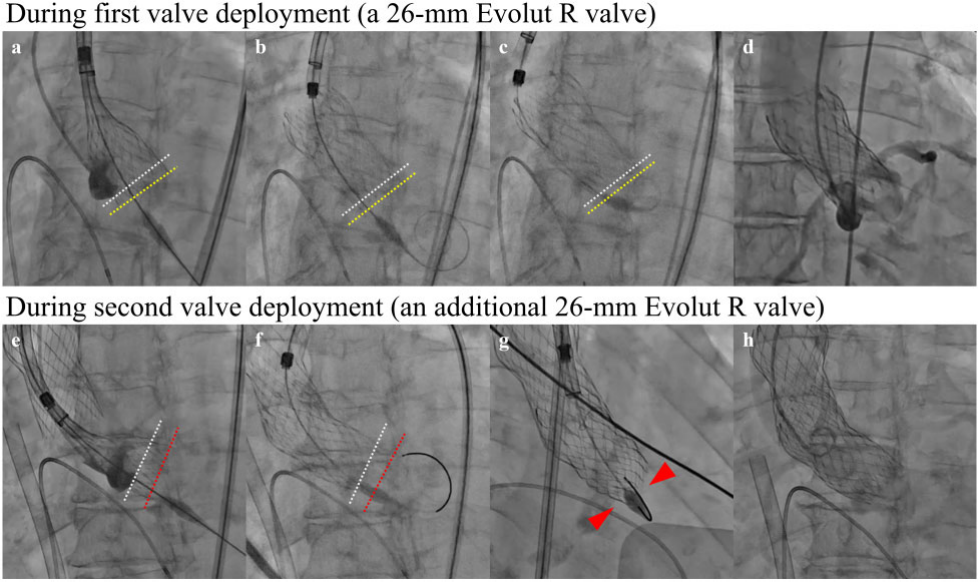
Fluoroscopy during the first and second valve deployments. (*A*) During 26-mm Evolut R valve (ERV) deployment. The white dotted lines show the native aortic annulus. The yellow dotted lines show the bottom of the ERV. (*B*) Immediately after the 26-mm ERV deployment in the proper position. (*C*) A delivery system (DS) of the ERV being caught in the ERV. (*D*) The ERV moving to the ascending aorta side. (*E*) During an additional 26-mm ERV deployment. The red dotted lines show the bottom of the second ERV. (*F*) Immediately after the second ERV deployment in the proper position. (*G*) The second ERV showing extreme under-expansion (red arrowheads). (*H*) The second ERV moving to the side of the ascending aorta.

**Figure 2:**
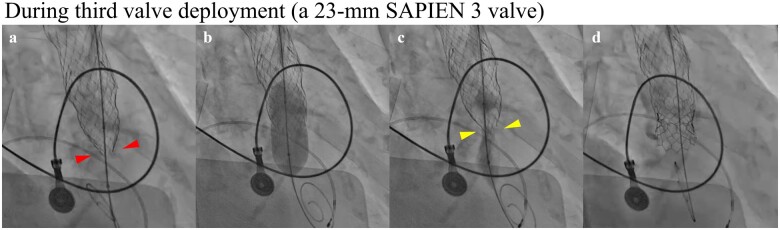
Fluoroscopy during the third valve deployment. (*A*) Before balloon dilation with a 20-mm balloon. The red arrowheads show extreme under-expansion of a 26-mm Evolut R valve (ERV). (*B*) During balloon dilation. (*C*) Immediately after balloon dilation. The yellow arrowheads show the stent recoil of the 26-mm ERV. (*D*) After a 23-mm SAPIEN 3 valve deployment.

Pre-procedural computed tomography (CT) measurements are shown in Supplementary material online, *Figure S1* and *Table 1*, and the aortic root size was considered sufficiently large to accommodate a TAVI. The aortic valve (AV) also had no massive calcification. However, scattered calcifications were noted in the non-coronary leaflet (*Video 1*), and the calcium volume (CV) index was considered high (Supplementary material online, *Figure S2*). Therefore, we selected a 26-mm Evolut R valve (ERV; Medtronic, Minneapolis, MN, USA) to avoid aortic root rupture (Supplementary material online, *Figure S3*). TAVI was performed under local anaesthesia via the transfemoral approach. After pre-dilation of the AV with a 16-mm balloon, a 26-mm ERV was deployed at the proper position (*Figure 1A* and *B*, Supplementary material online, *Figure S4*). However, resistance was felt when we attempted to remove the DS of the ERV (DS-ERV) from the ERV (*Figure 1C*, *Video 2*). Wire manipulation changed the DS-ERV position, suggesting that we could safely remove the DS-ERV. However, the ERV embolized to the ascending aorta while the DS-ERV was being removed from the ERV (*Figure 1D*, *Video 3*). Haemodynamic collapse, caused by aortic regurgitation or coronary malperfusion, occurred immediately after embolization. Therefore, emergency intubation was performed, and emergency veno-arterial extracorporeal membrane oxygenation (V-A ECMO) was percutaneously introduced via the left common femoral artery and venous routes as a precaution. After repositioning the ERV to the ascending aorta using a 6-Fr EN Snare (working diameter 12-20 mm; Merit Medical Systems, South Jordan, UT, USA), an additional 26-mm ERV was deployed slightly to the left ventricular side as compared to the initial position of the first ERV (*Figure 1E*). However, resistance was again felt when we attempted to remove the DS-ERV from the second ERV (*Figure 1F*). Thus, to evaluate expansion of the second ERV, we performed multi-directional fluoroscopy. The fluoroscopy revealed that the second ERV was extremely under-expanded (*Figure 1G*, Supplementary material online, *Video S1*), which caused catching of the DS-ERV in the second ERV. Manipulation of the wire and DS-ERV could not release this catching of the DS-ERV, which resulted in upward embolization of the second ERV (*Figure 1H*). Emergency open surgery for removal of the two embolized valves and surgical AV replacement was too risky for the patient because of the need for cardiopulmonary bypass, and the embolized valves did not float in the ascending aorta (Supplementary material online, *Figure S5*). Therefore, we decided to perform TAVI again; finally, a 23-mm SAPIEN 3 valve (Edwards Lifesciences, Irvine, CA, USA), which was a BEV, was successfully implanted with −2 mL under-filling following a pre-dilation with a 20-mm balloon (*Figure 2*, Supplementary material online, *Video S2*). The V-A ECMO was removed, and extubation was performed on the day after TAVI because the patient’s condition had improved. Single antiplatelet therapy using clopidogrel (75 mg/day) was also administered on the day after TAVI. The patient was discharged to home 9 days after TAVI without further complications. Our patient has survived beyond the 1-month follow-up with slight shortness of breath, classified as NYHA class II. Transthoracic echocardiography showed a normal LVEF (61%) with a mean pressure gradient of 10 mmHg and trivial paravalvular regurgitation. We will continue close follow-up observation.

**Table 1:** Pre-procedural computed tomography measurements of the aortic root

Annulus		LVOT area (mm^2^)	Sinus of Valsalva dimensions								STJ area (mm^2^)
Perimeter (mm)	Area (mm^2^)		Diameter (mm)			CV (mm^3^)				CV index (mm^3^/m^2^)	
66.9	346	315	NCC	RCC	LCC	NCC	RCC	LCC	Total	Total	433
			28.1	26.6	26.7	483	138	81	701	527	

CV, calcium volume; LCC, left coronary cusp; LVOT, left ventricular outflow tract; NCC, non-coronary cusp; RCC right coronary cusp; STJ, sino-tubular junction.

## Discussion

TAVE usually occurs during or shortly after valve deployment because of sizing errors, post-dilation, fast-rate pacing failure, and predisposing factors that include the use of SEVs and bicuspid aortic valves.^1^^,^^2^ In this case, we first selected an SEV because a BEV implantation was considered a risk factor for aortic root rupture due to the patient’s high CV index^4^^,^^5^ (Supplementary material online, *Figure S2*). The two ERVs were properly prepared before implantation (Supplementary material online, *Figure S6*, *Video S3*) and were implanted at appropriate positions as mentioned in Supplementary material online, *Figure S4*. Furthermore, as shown in *Videos 2* and 3, the ERVs did not move even when we pulled the DS-ERV with enough force that the DS was pulled to the lesser curvature of the aortic arch, and the stent frame deformation of the first ERV was fluoroscopically noted just before embolization to the ascending aorta. Additionally, multi-directional fluoroscopy revealed that the second ERV was extremely under-expanded, which was reportedly correlated with CV of the AVs.^6^ These findings suggested that catching of the DS in the ERV caused multiple valve embolizations in this case.

It is important to confirm the expansion of TAV using multi-directional fluoroscopy after valve deployment before DS-TAV removal to avoid TAVE in TAVI, especially when using an SEV. In this case, it was difficult to predict the extreme under-expansion of the ERVs because pre-procedural CT images showed no massive calcification on the AV. In addition, the ERVs seemed to have an adequate expansion at the angle where the ERVs were deployed.

**Figure 3:**
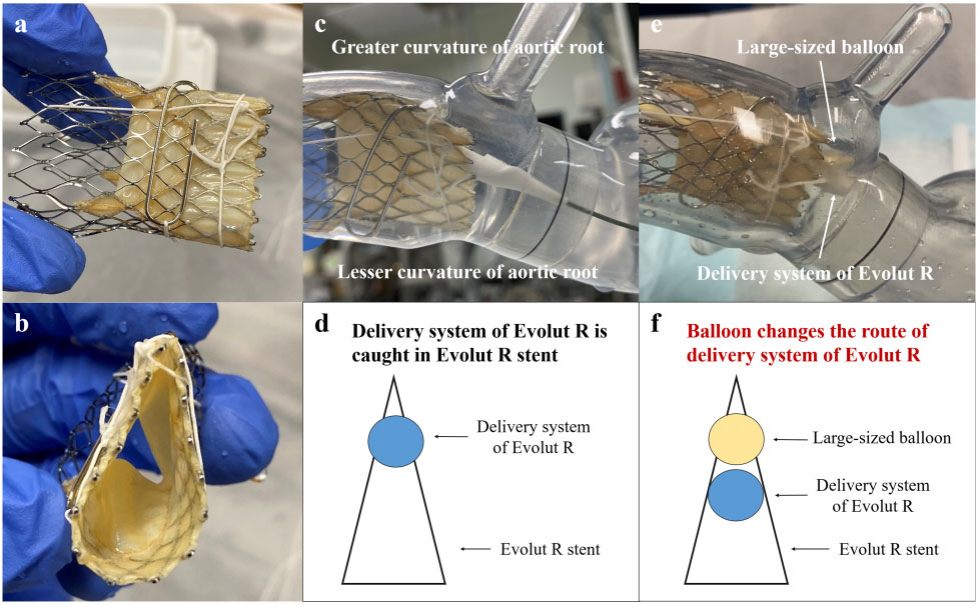
Images on the *in vitro* experiment. (*A* and *B*) A 26-mm Evolut R valve (ERV) expressing extreme under-expansion with thread and a clip. (*C*) Image and (*D*) schema of the delivery system (DS) of the ERV (DS-ERV) caught in the ERV. (*E*) Image and (*F*) schema of the releasing of the DS-ERV from the ERV using a large-sized balloon.

In case of catching of a DS-TAV in the TAV, the inflation of large-sized balloons where a DS-TAV was entrapped might help release the catch. In an *in vitro* experiment performed using a 26-mm ERV expressing extreme under-expansion, balloon inflation of a large-sized balloon at the catching point showed a possibility of releasing the DS-ERV catch by changing its position (*Figure 3*). Practically, the use of balloons for endovascular therapy may play a role similar to that of the large-sized balloon in the *in vitro* experiment. It seems difficult to perform this procedure *in vivo*, but it could be worth trying before TAVE, as TAVE could have a dismal prognosis.^1^^,^^3^

In conclusion, under-expansion of a TAV can cause TAVE due to catching of the DS-TAV in the TAV in TAVI. Confirming the expansion of a TAV using multi-directional fluoroscopy before removing DS-TAV is important to avoid TAVE, even if pre-procedural CT shows no massive calcification on the AV.

## Lead author biography

**Figure:**
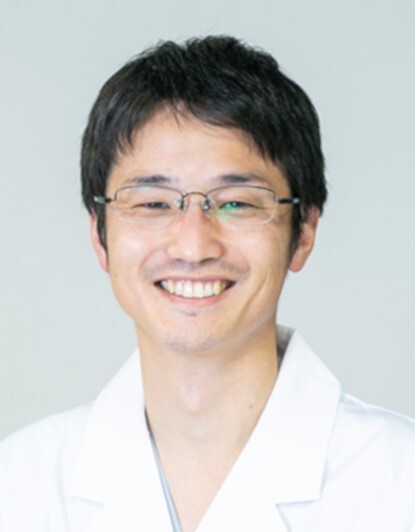


Masaki Tsuda is an interventional cardiologist. He graduated from Mie University and received the MD degree in 2010. He worked as a staff at Department of Cardiovascular Medicine, Osaka University, Japan from 2016 to 2019. From 2019, he serves as a staff cardiologist at Osaka Rosai Hospital, Japan.

## Supplementary material

Supplementary material is available at *European Heart Journal - Case Reports* online.

## Supplementary Material

ytaa497_Supplementary_Data
