# Supplementary material for: Implantation of three transcatheter aortic valves for embolization of two valves caused by under-expansion: a case report
Source: Eur Heart J Case Rep. 2020 Dec 15;5(1):ytaa497. doi: 10.1093/ehjcr/ytaa497 (PMC7898586; doi:10.1093/ehjcr/ytaa497)
Supplement: ytaa497_Supplementary_Data [file ytaa497_supplementary_data.zip › Figure_S2.pdf]

Fig. S2. The calcium volume of the patient was considered to be high, so we first selected a self-expandable valve.

A previous study reported that the high calcium volume index measured using computed tomography, which was defined as 517 >mm^3/m^2, was a risk factor for aortic root rupture (5). In this case, the aortic valve had no massive calcification. However, scattered calcifications were noted in the non-coronary leaflet, and the calcium volume index was calculated as 527 mm^3/m^2. Therefore, we considered the calcium volume of the patient as high, and we selected a 26-mm Evolut R valve to avoid aortic root rupture.
