# Supplementary material for: Implantation of three transcatheter aortic valves for embolization of two valves caused by under-expansion: a case report
Source: Eur Heart J Case Rep. 2020 Dec 15;5(1):ytaa497. doi: 10.1093/ehjcr/ytaa497 (PMC7898586; doi:10.1093/ehjcr/ytaa497)
Supplement: ytaa497_Supplementary_Data [file ytaa497_supplementary_data.zip › Figure_S3.pdf]

Fig. S3. The differences between self-expandable valves and balloon-expandable valves.

The differences between self-expandable valves (SEVs) and balloon-expandable valves (BEVs)

SEVs and BEVs differ in how they are implanted. The implantation of BEVs requires a balloon dilation under rapid pacing. On the other hand, SEVs expand themselves following gradual unsheathing because SEVs include nitinol. Therefore, SEVs require neither balloon dilation nor rapid pacing.

The most common reason for selecting either

The use of BEVs is reported one of the risk factors for aortic root rupture, which is a catastrophic complication of transcatheter aortic valve implantation (TAVI). Therefore, in cases of TAVI with massive calcification on the aortic valve or left ventricular outflow tract, the implantation of SEVs is safer than that of BEVs because SEV implantation is less likely to cause aortic root rupture due to how it expands (4).
