# Supplementary material for: Implantation of three transcatheter aortic valves for embolization of two valves caused by under-expansion: a case report
Source: Eur Heart J Case Rep. 2020 Dec 15;5(1):ytaa497. doi: 10.1093/ehjcr/ytaa497 (PMC7898586; doi:10.1093/ehjcr/ytaa497)
Supplement: ytaa497_Supplementary_Data [file ytaa497_supplementary_data.zip › Figure_S4.pdf]

Fig. S4. The anatomical landmarks for appropriate positioning of the transcatheter aortic valve

It is used for appropriating positioning of an Evolut R valve (ERV) that a distance of 3-5 mm between the stent edge of an ERV and the bottom of the non-coronary cusp. In this case, the distance was 3.5 mm. Therefore, we considered that the two ERVs were implanted at appropriate positions.
