# Supplementary material for: Implantation of three transcatheter aortic valves for embolization of two valves caused by under-expansion: a case report
Source: Eur Heart J Case Rep. 2020 Dec 15;5(1):ytaa497. doi: 10.1093/ehjcr/ytaa497 (PMC7898586; doi:10.1093/ehjcr/ytaa497)
Supplement: ytaa497_Supplementary_Data [file ytaa497_supplementary_data.zip › Figure_S5.pdf]

Fig. S5. The eventuality of the two embolised valves.

The two embolised valves might cause complications, including cerebral infarction by scattering of mural thrombosis or plaque owing to the floating embolised valves in the ascending aorta.
