# Supplementary material for: Implantation of three transcatheter aortic valves for embolization of two valves caused by under-expansion: a case report
Source: Eur Heart J Case Rep. 2020 Dec 15;5(1):ytaa497. doi: 10.1093/ehjcr/ytaa497 (PMC7898586; doi:10.1093/ehjcr/ytaa497)
Supplement: ytaa497_Supplementary_Data [file ytaa497_supplementary_data.zip › Figure_S6.pdf]

Fig. S6. How to mount the two Evolut R valves on the each delivery system, and fluoroscopy images during confirming adequate preparation of the valves.

We gradually mounted the two ERVs on the each DS *ex vivo* while visually checking for no kink of the stent frame of the ERVs; we fluoroscopically confirmed their correct preparation before implantation as below:

Fluoroscopy images during confirming preparation of (a) the first ERV and (b) the second ERV

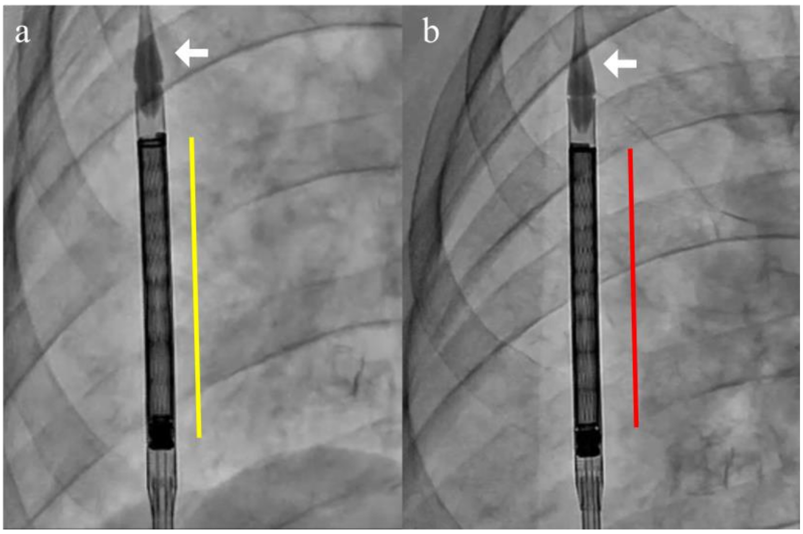

The yellow and red line showed the fully sheathed first ERV and the fully sheathed second ERV, respectively. The valves were stored in the each sheath without kink of the stent of the ERVs. The white arrows indicated the 'nose corn' of the DSs of the ERVs.
