# Supplementary figures and images for: Implantation of three transcatheter aortic valves for embolization of two valves caused by under-expansion: a case report
Source: Eur Heart J Case Rep. 2020 Dec 15;5(1):ytaa497. doi: 10.1093/ehjcr/ytaa497 (PMC7898586; doi:10.1093/ehjcr/ytaa497)

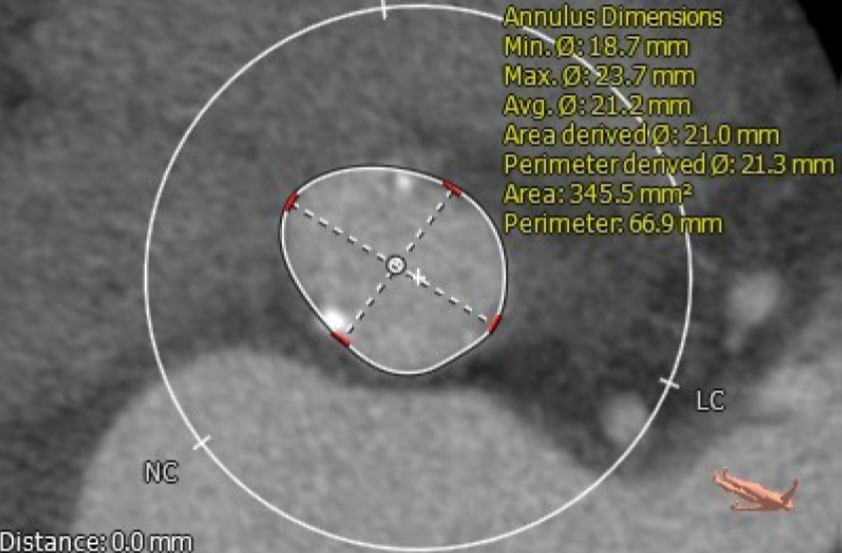

Supplement: ytaa497_Supplementary_Data [file ytaa497_supplementary_data.zip › Figure_S1.tif]
